# Thermal Expansion in Layered Na_*x*_MO_2_

**DOI:** 10.1038/s41598-018-22279-9

**Published:** 2018-03-05

**Authors:** Wataru Kobayashi, Ayumu Yanagita, Takahiro Akaba, Takahiro Shimono, Daiki Tanabe, Yutaka Moritomo

**Affiliations:** 1 Graduate School of Pure and Applied Sciences, University of Tsukuba, Ibaraki, 305-8571 Japan; 2 Division of Physics, Faculty of Pure and Applied Sciences, University of Tsukuba, Ibaraki, 305-8571 Japan; 3 Tsukuba Research Center for Energy Materials Science (TREMS), University of Tsukuba, Ibaraki, 305-8571 Japan

## Abstract

Layered oxide Na_*x*_MO_2_ (M: transition metal) is a promising cathode material for sodium-ion secondary battery. Crystal structure of O3- and P2-type Na_*x*_MO_2_ with various M against temperature (*T*) was systematically investigated by synchrotron x-ray diffraction mainly focusing on the *T*-dependences of *a*- and *c*-axis lattice constants (*a* and *c*) and *z* coordinate (*z*) of oxygen. Using a hard-sphere model with minimum Madelung energy, we confirmed that *c*/*a* and *z* values in O3-type Na_*x*_MO_2_ were reproduced. We further evaluated the thermal expansion coefficients (*α*_*a*_ and *α*_*c*_) along *a*- and *c*-axis at 300 K. The anisotropy of the thermal expansion was quantitatively reproduced without adjustable parameters for O3-type Na_*x*_MO_2_. Deviations of *z* from the model for P2-type Na_*x*_MO_2_ are ascribed to Na vacancies characteristic to the structure.

## Introduction

**Figure 1:**
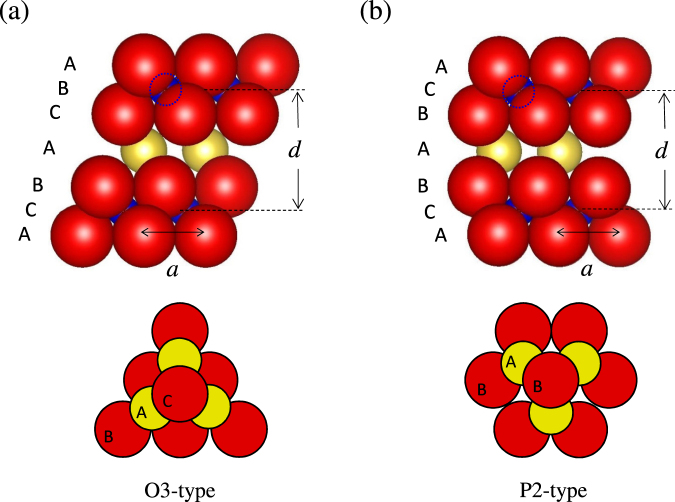
Schematic figure of (**a**) O3- and (**b**) P2-NaMO_2_ structure. Red, yellow, and blue spheres represent O, Na, and M (transition metal element), respectively. M is sandwiched by upper and lower oxygen layers. In the P2-type structure, only the Na1 site [the atomic coordinates of Na1 are $(\frac{1}{3},\frac{2}{3},\frac{3}{4})$] is shown. The bottom panels show top views of O-Na-O stackings in the O3- and P2-type structures, respectively. The BAC stacking in O3 forms NaO_6_ octahedron, and the BAB stacking in P2 forms NaO_6_ triangular prism.

Sodium-ion-secondary battery (SIB) stores electrochemical energy through Na^+^ intercalation/deintercalation process. Due to large Clark number (=2.63) of Na compared with that (=0.006) of Li, SIBs can be a promising next-generation battery for storage of natural energy at a power plant and for a large-scale device such as electrical vehicle. Layered oxide Na_*x*_MO_2_ (M: transition metal) is a typical cathode material for SIBs^1–3^. Crystal structure of this material is categorized into two typical structures: O3 and P2 types^4^. Figure 1 shows schematic structures of (a) O3-type and (b) P2-type NaMO_2_. Red, yellow, and blue spheres represent O, Na, and M, respectively. M is surrounded by six oxygens, and a MO_6_ octahedron is formed. The edge-sharing MO_6_ octahedra form a MO_2_ layer. Both O3- and P2-type NaMO_2_ exhibit alternately stacked MO_2_ layers and Na sheets. The sodium sheet, upper and lower oxygen sheets stack as BAC resulting in the octahedral Na site. In the P2-type NaMO_2_, the sodium and oxygen sheets stack as BAB resulting in the prismatic Na site. O3-type NaMO_2_ (M = Ti, Cr, Mn, Co, Ni) and P2-type Na_*x*_MO_2_ (M = Mn and Co) were found to exhibit Na^+^ intercalation/deintercalation in early 1980s^5–9^. Concerning the discovery of hard carbon (≥200 mAh/g) as anode material of SIB^10^, electrochemical properties of Na_*x*_MO_2_ are extensively reported^11–20^. Very recently, substitution effects on the battery properties in O3-type structure^21–27^ and P2-type structure^28–45^ were extensively studied to reduce expensive element and improve the cyclability and capacity.

Not only electrochemical properties but also superconductivity^46^, crystal structure^47–57^, magnetism^58–61^, thermoelectric effect^62,63^, and first-principle calculation^64–67^ of the end family are also studied. Fujita *et al*. found that Na_*x*_CoO_2−*δ*_ single crystal shows a large dimensionless figure-of-merit of *ZT* = 1 at 800 K^63^, which has motivated practical use for waste heat recovery at high temperatures (*T*). An isostructural O3-type LiMO_2_ is widely used as a cathode material in lithium-ion-secondary battery (LIB)^68,69^. This family is also studied as a thermoelectric material^70^ and a cathode material of solid oxide fuel cell (SOFC)^71^. In particular, Lan and Tao found that Li_*x*_Al_0.5_Co_0.5_O_2_ shows good proton conductivity of 0.1 Scm^−1^ at 773 K^71^, which is the highest among those of known polycrystalline proton-conducting materials. During operation of energy devices such as LIB(SIB), thermoelectric device, and SOFC, these materials are exposed to a variation and/or a gradient of temperature. A mismatch in thermal expansion coefficients in between the components can result in high stresses around the interface leading to deterioration of the device. Thus, evaluation and systematical comprehension of thermal expansion behaviors in this class of materials are important.

In this paper, we report systematic structural analysis of four O3- and five P2-type Na_*x*_MO_2_ samples against *T* (300 K ≤ *T* ≤ 800 K) performed by synchrotron x-ray diffraction focusing on thermal expansion. To understand the thermal expansion behavior, we constructed a hard-sphere model with constraint that M, upper and lower oxygens are connected each other. We confirmed that the calculated *d*/*a* [*d*: interlayer distance, *a*: *a*-axis lattice constant] and *z* well reproduced the experimental values for O3-type Na_*x*_MO_2_. By introducing *T*-linear expansion of the hard sphere, the anisotropy of the thermal expansion was quantitatively reproduced without adjustable parameter for O3-type Na_*x*_MO_2_.

## Results

### Temperature dependence of *a*(*c*)-axis lattice constants and *z* coordinate of oxygen

**Figure 2:**
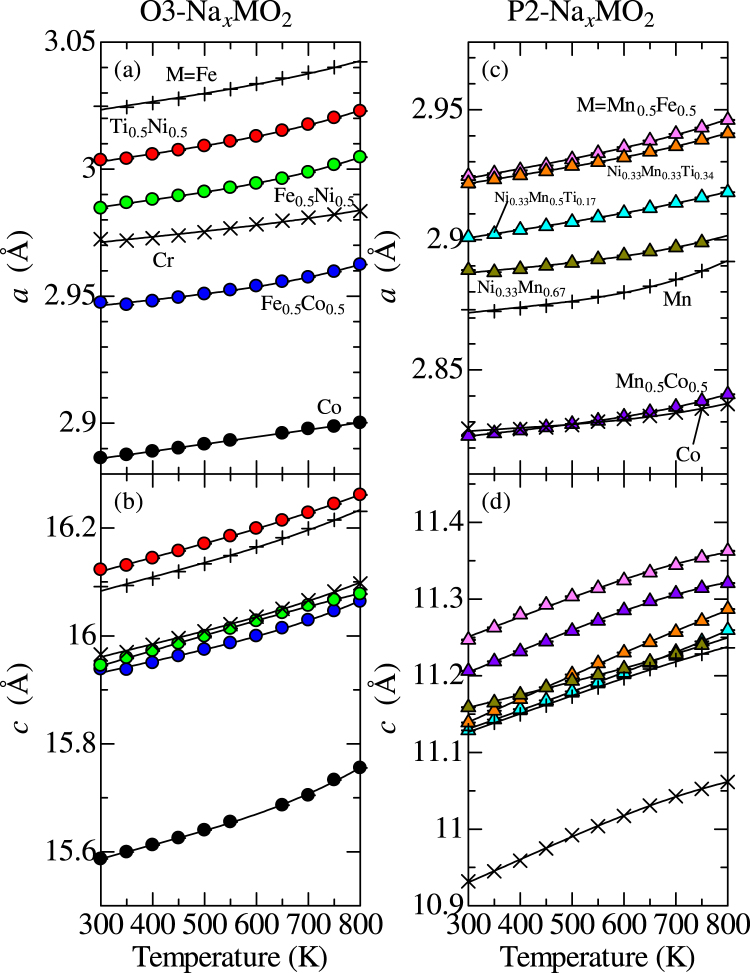
Temperature (*T*) dependence of (**a**) *a*-axis, (**b**) *c*-axis lattice constants (*a* and *c*) of O3-type Na_*x*_MO_2_. *T* dependence of (**c**) *a*, and (**d**) *c* of P2-type Na_*x*_MO_2_. The solid line represents a least-square fitting with use of a degree 3 polynomial function for *a* and *c*. *a* and *c* values of O3-Na_0.99_CrO_2_, O3-Na_0.99_FeO_2_, P2-Na_0.52_MnO_2_, and P2-Na_0.59_CoO_2_ were referred from our previous reports^56,57^.

Figure 2(a) and (b) show *a*-axis lattice constant (*a*), and (b) *c*-axis lattice constant (*c*) of O3-type Na_0.99_CrO_2_, Na_0.99_FeO_2_, Na_1.00_CoO_2_, Na_0.98_Fe_0.5_Co_0.5_O_2_, Na_0.99_Fe_0.5_Ni_0.5_O_2_, and Na_0.94_Ti_0.5_Ni_0.5_O_2_ against *T*. With *T*, *a* and *c* monotonically increase. Raw x-ray diffraction data and results of Rietveld refinements at 300 K are shown in Figs S1–S3. The solid line represents a least-square fitting with use of a degree 3 polynomial function. By using ${\alpha }_{a(c)}=\frac{d\,\mathrm{ln}\,a(c)}{dT}$, a linear thermal expansion coefficient along *a*- and *c*-axis was evaluated. Figure 2(c) and (d) show *a* and *c* of P2-type Na_0.52_MnO_2_, Na_0.59_CoO_2_, Na_0.50_Mn_0.5_Co_0.5_O_2_, Na_0.70_Ni_0.33_Mn_0.67_O_2_, Na_0.69_Ni_0.33_Mn_0.5_Ti_0.17_O_2_, Na_0.70_Ni_0.33_Mn_0.33_Ti_0.34_O_2_, and Na_0.48_Mn_0.5_Fe_0.5_O_2_. The P2-type compounds also show monotonical *T*-dependences of *a* and *c*. In Table 1, the values of *a*, *c* at 300 K, *α*_*a*_, and *α*_*c*_ of O3- and P2-type Na_*x*_MO_2_ at 300 K were listed.

**Table 1:** *a*-axis, *c*-axis lattice constants (*a* and *c*), *z* coordinate of oxygen (*z*) at 300 K, the linear thermal expansion coefficient [*α*_*a*_ (*α*_*c*_)] along *a*(*c*)-axis, and *α*_*c*_/*α*_*a*_ of O3- and P2-type Na_*x*_MO_2_ at 300 K.

Compound	*a* (Å)	*c* (Å)	*z*	*α*_*a*_ (10^−5^ K^−1^)	*α*_*c*_ (10^−5^ K^−1^)	*α*_*c*_/*α*_*a*_
^56^O3-Na_0.99_CrO_2_	^†^ 2.97247(3)	^†^15.96540(18)	0.23213(13)	^*^0.73	^*^1.52	^*^2.07
^56^O3-Na _0.99_ FeO _2_	^†^3.02477(2)	^†^16.09135(10)	0.23389(12)	^*^1.07	^*^1.63	^*^1.52
O3-Na_1.00_CoO_2_	2.88627(2)	15.58680(12)	0.23046(9)	0.98	1.71	1.74
O3-Na_0.98_Fe_0.5_Co_0.5_O_2_	2.94748(3)	15.93844(23)	0.23238(12)	0.79	1.37	1.73
O3-Na_0.99_Fe_0.5_Ni_0.5_O_2_	2.98463(9)	15.94545(63)	0.23334(17)	1.01	1.76	1.74
O3-Na_0.94_Ti_0.5_Ni_0.5_O_2_	3.00358(4)	16.12273(24)	0.23382(13)	1.06	1.61	1.51
^57^P2-Na_0.52_MnO_2_	^†^2.87311(9)	^†^11.1287(5)	0.0858(3)	^*^0.81	^*^2.11	^*^2.59
^57^P2-Na_0.59_CoO_2_	^†^2.82748(4)	^†^10.9319(2)	0.0881(3)	^*^0.48	^*^2.70	^*^5.61
P2-Na_0.50_Mn_0.5_Co_0.5_O_2_	2.82459(5)	11.20617(33)	0.08704(24)	0.84	2.42	2.87
P2-Na_0.70_Ni_0.33_Mn_0.67_O_2_	2.88835(5)	11.15881(29)	0.09120(28)	0.67	1.51	2.26
P2-Na_0.69_Ni_0.33_Mn_0.5_Ti_0.17_O_2_	2.90101(3)	11.12868(16)	0.09263(17)	1.07	2.20	2.05
P2-Na_0.70_Ni_0.33_Mn_0.33_Ti_0.34_O_2_	2.92173(4)	11.13896(22)	0.09381(21)	1.12	2.72	2.42
P2-Na_0.48_Mn_0.5_Fe_0.5_O_2_	2.92452(8)	11.24690(48)	0.08592(32)	1.30	2.26	1.74

^†^The original data were referred from previous reports^56,57^. ^*^α_*a*_ and *α*_*c*_ were reevaluated in a *T*-range of 300–800 K.

**Figure 3:**
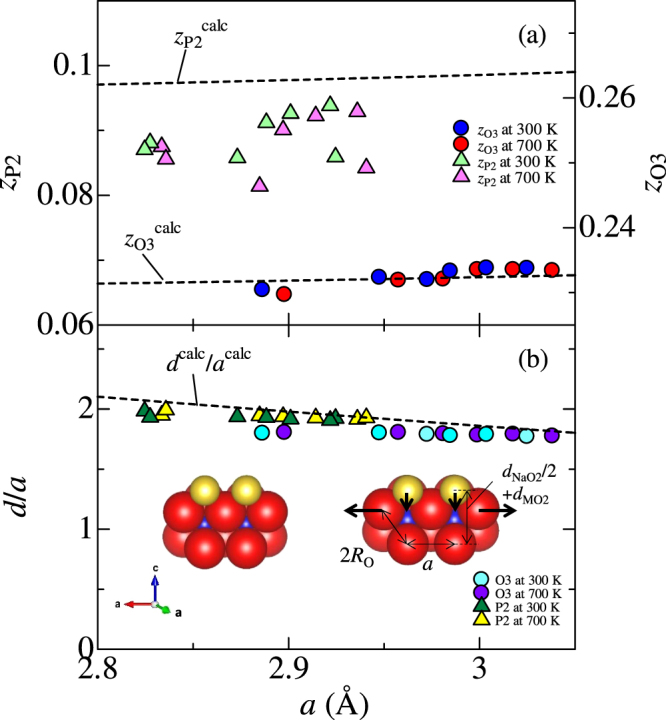
(**a**) *z* coordinate of oxygen for O3- (*z*_O3_) and P2-type (*z*_P2_) structure against *a*-axis lattice constant (*a*). Blue and red circles represent *z*_O3_ at 300 and 700 K, respectively. Light green and pink triangles represent *z*_P2_ at 300 and 700 K, respectively. The broken lines represent *z* calculated by the hard sphere model with minimum Madelung energy for O3 and P2-type compounds (${z}_{{\rm{O}}3}^{{\rm{calc}}}$ and ${z}_{{\rm{P}}2}^{{\rm{calc}}}$) against *a*, respectively. (**b**) The ratio of interlayer distance (*d*) to *a* against *a*. Light blue and purple circles represent *d*/*a* of O3-type compounds at 300 and 700 K, respectively. Green and yellow triangles represent *d*/*a* of P2-type compounds at 300 and 700 K, respectively. The broken line represents *d*/*a* calculated by the hard sphere model with minimum Madelung energy (*d*^calc^/*a*^calc^) against *a*. The inset of Fig. 3(b) shows a schematic view of the local atomic configuration around M.

Figure 3(a) shows *z* coordinate of oxygen for O3- and P2-type structure (*z*_O3_ and *z*_P2_) against *a* at 300 and 700 K. Blue and red circles represent *z*_O3_ at 300 and 700 K, respectively. Light green and pink triangles represent *z*_P2_ at 300 and 700 K, respectively. The values were almost independent of *T*. Figure 3(b) shows a ratio of interlayer distance (*d*) to *a* against *a* at 300 K, where *d* is *c*/3 for O3-type and *c*/2 for P2-type structure. Light blue and purple circles represent *d*/*a* of O3-type compounds at 300 and 700 K, respectively. Green and yellow triangles represent *d*/*a* of P2-type compounds at 300 and 700 K, respectively. *d*/*a* slightly decreases with an increase in *a*.

### Thermal expansion coefficients

**Figure 4:**
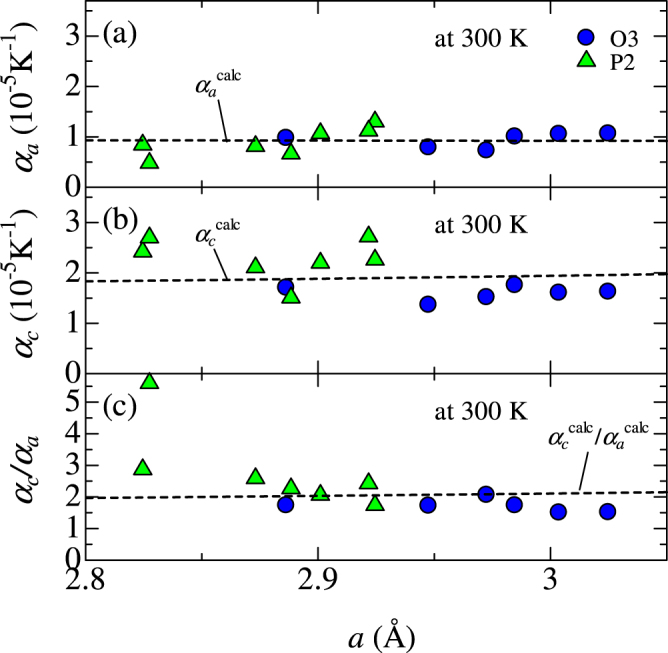
Linear thermal expansion coefficient (**a**) along *a*-axis (*α*_*a*_), (**b**) along *c*-axis (*α*_*c*_), and (**c**) *α*_*c*_/*α*_*a*_ against *a*-axis lattice constant (*a*). The broken lines in Fig. 4(a,b and c) represent *α*_*a*_, *α*_*c*_, and *α*_*c*_/*α*_*a*_ against *a* calculated by the hard-sphere model with minimum Madelung energy (${\alpha }_{a}^{{\rm{calc}}}$, ${\alpha }_{c}^{{\rm{c}}{\rm{a}}{\rm{l}}{\rm{c}}}$, and ${\alpha }_{c}^{{\rm{c}}{\rm{a}}{\rm{l}}{\rm{c}}}$/${\alpha }_{a}^{{\rm{calc}}}$).

Figure 4 shows (a) *α*_*a*_, (b) *α*_*c*_, and (c) *α*_*c*_/*α*_*a*_ against *a*. *α*_*a*_ and *α*_*c*_ of O3-type Na_1.00_CoO_2_ were 0.98 × 10^−5^ K^−1^ and 1.71 × 10^−5^ K^−1^, respectively. These values are comparable to those of inorganic compounds; *α*_*a*_ = 1.44 × 10^−5^ K^−1^ for LiMn_2_O_4_^72^, and *α*_*a*_(*α*_*c*_) = 0.85(2.5) × 10^−5^ K^−1^ for layered BaFe_1.84_Co_0.16_As_2_^73^. *α*_*a*_(*α*_*c*_) in Fig. 4 is rather scattered against *a* around the averaged value of 0.92(1.96) × 10^−5^ K^−1^. The ratio *α*_*c*_/*α*_*a*_ is also scattered around the averaged value (=2.30). However, the data point for P2-Na_0.59_CoO_2_ are seriously deviated from the average value. This is probably due to the Na ordering^67^.

## Discussion

### A hard-sphere model with minimum Madelung energy

We have constructed a hard-sphere structural model for O3- and P2-type NaMO_2_ to reproduce the experimental results (*d*/*a*, *z*, *α*_*c*_/*α*_*a*_). Firstly, imagine a sheet consists of hard spheres that were arrayed on triangular lattice, and then the sheet is alternately stacked as shown in Fig. 1. In the model, hard spheres of Na, M, and O were assumed to have Shannon’s ionic radius^74^; *R*_Na_ = 1.02 Å, *R*_O_ = 1.40 Å, and *R*_M_ is a variable parameter that takes 0.58–0.7 Å, respectively. Since the ionic radius of the hard sphere is different from one another, the structure can not be the hexagonal close-packed structure, and several structures with different *a* are possible for the unique value of *R*_M_. Here, we adopted a constraint that M, upper, and lower oxygens coroneted each other, because the constraint minimizes the Madelung energy against *a* (vide infra). For a calculation of thermal expansion coefficient, the hard sphere is assumed to expand in proportion to the temperature difference (Δ*T*). Δ*T* dependence of *R*_Na_ is expressed as ${R}_{{\rm{Na}}}({\rm{\Delta }}T)={R}_{{\rm{Na}}}+A{m}_{{\rm{Na}}}^{-1}{R}_{{\rm{Na}}}{\rm{\Delta }}T$, where *m*_N*a*_(=22.99) is atomic weight of Na atom. Similarly, Δ*T* dependences of *R*_O_ and *R*_M_ are expressed as ${R}_{{\rm{O}}}({\rm{\Delta }}T)={R}_{{\rm{O}}}+A{m}_{{\rm{O}}}^{-1}{R}_{{\rm{O}}}{\rm{\Delta }}T$, and ${R}_{{\rm{M}}}({\rm{\Delta }}T)={R}_{{\rm{M}}}+A{m}_{{\rm{M}}}^{-1}{R}_{{\rm{M}}}{\rm{\Delta }}T$, where *m*_O_(=16.00) and *m*_M_(=55.85) (We used 55.85 of the atomic weight of Fe as *m*_M_ although M is not only Fe but also mixture of Ti, Mn, Fe, and Co. When we used 47.88 of the atomic weight of Ti as *m*_M_, the calculated *α*_c_/*α*_a_ worse reproduces the experiments.) are atomic weights of O and M, respectively.

Now, let us derive the expression (*a*^calc^) of *a*-axis lattice constant as a function of Δ*T*. Note that the in-plane nearest-neighbor oxygen distance is *a* for both the P2- and O3-structure. Considering the above-mentioned constraint, *a*^calc^(Δ*T*) for both O3- and P2-structures is expressed as

1 $${a}^{{\rm{calc}}}({\rm{\Delta }}T)=2\sqrt{{[{R}_{{\rm{O}}}({\rm{\Delta }}T)+{R}_{{\rm{M}}}({\rm{\Delta }}T)]}^{2}-{R}_{{\rm{O}}}{({\rm{\Delta }}T)}^{2}}\mathrm{.}$$

This equation is easily derived using Pythagorean theorem. As shown in Eq. 1, *a*^calc^ strongly depends on *R*_M_ value. Due to the finite ionic radius of oxygen (*R*_O_ = 1.40 Å), minimum value of *a*^calc^ is 2.80 Å. At *a*^calc^ = 2.80 Å, *R*_M_ is evaluated to be ≈0.5799 Å using Eq. 1. With an increase in *R*_M_, the oxygen triangular lattice expands in order to keep the connection between M, upper and lower oxygens.

Na sheet is sandwiched by the MO_2_ layers as BAC (BAB) in the O3-type (P2-type) structure. We noted that the expression (*d*^calc^) of interlayer distance is independent of the stacking manner, and the Δ*T*-dependence of *d*^calc^ for O3- and P2-type structure is expressed as,

2 $${d}^{{\rm{calc}}}({\rm{\Delta }}T)=\sqrt{{\mathrm{[2}{R}_{{\rm{O}}}({\rm{\Delta }}T)]}^{2}-\frac{{a}^{{\rm{calc}}}{({\rm{\Delta }}T)}^{2}}{3}}+2\sqrt{{[{R}_{{\rm{O}}}({\rm{\Delta }}T)+{R}_{{\rm{Na}}}({\rm{\Delta }}T)]}^{2}-\frac{{a}^{{\rm{calc}}}{(\Delta T)}^{2}}{3}}\mathrm{.}$$

The first and the second terms correspond to ${d}_{{{\rm{MO}}}_{2}}^{{\rm{calc}}}$ and ${d}_{{{\rm{NaO}}}_{2}}^{{\rm{calc}}}$, where ${d}_{{{\rm{MO}}}_{2}}^{{\rm{calc}}}$ and ${d}_{{{\rm{NaO}}}_{2}}^{{\rm{calc}}}$ are the thicknesses of MO_2_ and NaO_2_ layers, respectively. A relationship between *d*^calc^ and the expression of *c* (*c*^calc^) is expressed as $3{d}^{{\rm{calc}}}({\rm{\Delta }}T)={c}_{{\rm{O}}3}^{{\rm{calc}}}({\rm{\Delta }}T)$ and $2{d}^{{\rm{calc}}}({\rm{\Delta }}T)={c}_{{\rm{P}}2}^{{\rm{calc}}}({\rm{\Delta }}T)$ for O3- and P2-type structures. By using Pythagorean theorem, Eq. 2 is easily derived. Expressions of *z* (*z*^calc^) for O3- and P2-type structures are derived as

3 $${z}_{{\rm{O}}3}^{{\rm{calc}}}({\rm{\Delta }}T)=\frac{1}{6}+\frac{1}{6{d}^{{\rm{c}}alc}({\rm{\Delta }}T)}\sqrt{{\mathrm{[2}{R}_{{\rm{O}}}({\rm{\Delta }}T)]}^{2}-\frac{{a}^{{\rm{calc}}}{({\rm{\Delta }}T)}^{2}}{3}},$$

and

4 $${z}_{{\rm{P}}2}^{{\rm{calc}}}({\rm{\Delta }}T)=\frac{1}{4{d}^{{\rm{calc}}}({\rm{\Delta }}T)}\sqrt{{\mathrm{[2}{R}_{{\rm{O}}}({\rm{\Delta }}T)]}^{2}-\frac{{a}^{{\rm{calc}}}{({\rm{\Delta }}T)}^{2}}{3}},$$

respectively.

**Figure 5:**
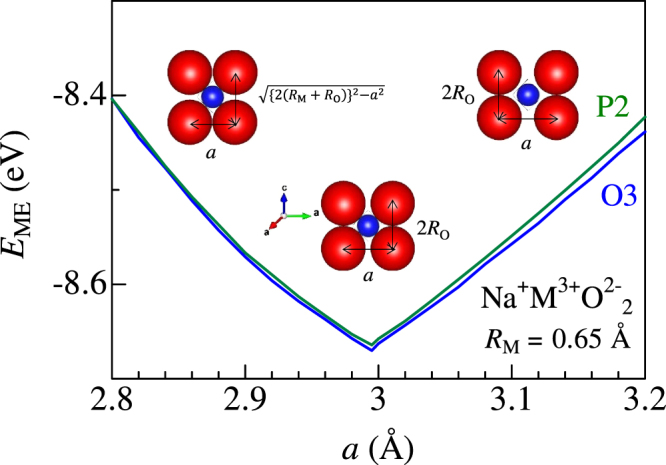
Madelung energy (*E*_ME_) of O3- and P2-type NaMO_2_ against *a* for the hard-sphere model. *R*_M_ was fixed at 0.65 Å.

Now, let us consider the stability of the hard-sphere model with the constraint that M, upper and lower oxygens coroneted to each other. For this purpose, we calculated the Madelung energy at a specific *R*_M_ (=0.65 Å) against *a*. We show that this model exhibits minimum Madelung energy (*E*_ME_). Figure 5 shows *E*_ME_ of O3- and P2-type NaMO_2_ against *a*. Our constraint gives *a*^calc^ ≈ 2.995 Å [Eq. 1] at *R*_M_ = 0.65 Å. With the O3 structure, the *a*^calc^ value corresponds to the minimum position of *E*_ME_ (−8.67 eV). With an increase in *a* from 2.995 Å, M becomes isolated from the surrounding oxygens (the right-side inset of Fig. 5), and *E*_ME_ increases (Note that the oxygen positions were controlled by Eqs 3 and 4). With an decrease in *a* from 2.995 Å, the upper and lower oxygens are separated (the left-side inset of Fig. 5), and *E*_ME_ increases as well (We used ${d}^{{\rm{calc}}}\mathrm{=2}\sqrt{{({R}_{{\rm{M}}}+{R}_{{\rm{O}}})}^{2}-\frac{{a}^{2}}{3}}+2\sqrt{{[{R}_{{\rm{O}}}+{R}_{{\rm{Na}}}]}^{2}-\frac{{a}^{2}}{3}}$, ${z}_{{\rm{O}}3}^{{\rm{calc}}}=\frac{1}{6}+\frac{1}{6{d}^{{\rm{calc}}}}\sqrt{{({R}_{{\rm{M}}}+{R}_{{\rm{O}}})}^{2}-\frac{{a}^{2}}{3}}$, and ${z}_{{\rm{P}}2}^{{\rm{calc}}}=\frac{1}{4{d}^{{\rm{calc}}}}\sqrt{{({R}_{{\rm{M}}}+{R}_{{\rm{O}}})}^{2}-\frac{{a}^{2}}{3}}$ for the calculation below *a* = 2.995 Å). Similar results are obtained for the P2 structure. Thus, our model is energetically stable against the variation of *a*. Our constraint that M, upper and lower oxygens connected to each other causes the compact layered structure and minimized the long-range Coulomb energy between the layers. We call our model “hard-sphere model with minimum Madelung energy”.

### Companion of the structural parameters and the thermal expansion coefficients with the model

The broken line in Fig. 3(b) is the calculated *d*/*a* based on the hard-sphere model with minimum Madelung energy (see Eqs 1 and 2). *d*^calc^/*a*^calc^ decreases with an increase in *a*^calc^, which reproduces experimental results. The decrease in *d*^calc^/*a*^calc^ is schematically depicted in the inset of Fig. 3(b). When O-O distance along in-plane direction elongates due to increase in *R*_M_, Na atoms relatively sink down along out-of-plane direction. (Decrease in ${d}_{{{\rm{NaO}}}_{2}}\mathrm{/(2}a)$ against *a* is displayed in Fig. S5). We further calculated *z* and plotted them in Fig. 3(a). In the O3-type compounds, *z*^calc^ well reproduces the experimental data. In the P2-type compounds, however, *z*^calc^ is slightly smaller than ${z}_{{\rm{P}}2}^{{\rm{calc}}}$. We ascribed the smaller *z*_P2_ to the Na vacancies characteristic to the P2 structure. With the vacancies, the nominal valence of M became higher, and hence *R*_M_ becomes smaller. Our model tells us that ${z}_{{\rm{P}}2}^{{\rm{calc}}}$ becomes smaller if *R*_M_ becomes smaller.

The broken lines in Fig. 4 represent the calculated *α*_*a*_, *α*_*c*_, and *α*_*c*_/*α*_*a*_ (${\alpha }_{a}^{{\rm{calc}}}$, ${\alpha }_{{c}}^{{\rm{calc}}}$, and ${\alpha }_{c}^{{\rm{calc}}}$/${\alpha }_{a}^{{\rm{c}}{\rm{a}}{\rm{l}}{\rm{c}}}$), respectively. ${\alpha }_{a}^{{\rm{c}}{\rm{a}}{\rm{l}}{\rm{c}}}$(${\alpha }_{c}^{{\rm{calc}}}$) was evaluated by using the equation, $\frac{d\,\mathrm{ln}\,{a}^{{\rm{calc}}}({\rm{\Delta }}T)}{d{\rm{\Delta }}T}$ $[\frac{d\,\mathrm{ln}\,{c}^{{\rm{calc}}}({\rm{\Delta }}T)}{d\Delta T}]$. The only fitting parameter *A* (=2.56 × 10^−4^ K^−1^) was chosen to fit the average value of *α*_*a*_ (=0.92 × 10^−5^ K^−1^). Using the same value of *A*, ${\alpha }_{c}^{{\rm{calc}}}$ was found to reproduce the magnitude of experimental value for the O3 materials. On the other hand, larger *α*_*c*_ for the P2 materials is possibly due to Na vacancies except for the data point of Na_0.59_CoO_2_. We note that $\frac{{\alpha }_{c}^{{\rm{calc}}}}{{\alpha }_{a}^{{\rm{calc}}}}$ is expressed without the adjustable parameter *A*,

5 $$\begin{array}{rcl}{\tfrac{{\alpha }_{c}^{{\rm{calc}}}}{{\alpha }_{a}^{{\rm{calc}}}}|}_{{\rm{\Delta }}T=0} & = & \tfrac{\tfrac{2{m}_{{\rm{O}}}^{-1}{R}_{{\rm{O}}}^{2}}{{m}_{{\rm{M}}}^{-1}{R}_{{\rm{M}}}{R}_{{\rm{O}}}+({m}_{{\rm{O}}}^{-1}{R}_{{\rm{O}}}+{m}_{{\rm{M}}}^{-1}{R}_{{\rm{M}}}){R}_{{\rm{M}}}}-\tfrac{2}{3}}{4\sqrt{\{{(\tfrac{2{R}_{{\rm{O}}}}{{a}_{0}})}^{2}-\tfrac{1}{3}\}\{{(\tfrac{{R}_{{\rm{O}}}+{R}_{{\rm{Na}}}}{{a}_{0}})}^{2}-\tfrac{1}{3}\}}+2\{{(\tfrac{2{R}_{{\rm{O}}}}{{a}_{0}})}^{2}-\tfrac{1}{3}\}}\\  &  & +\tfrac{\tfrac{({m}_{{\rm{O}}}^{-1}{R}_{{\rm{O}}}+{m}_{{\rm{Na}}}^{-1}{R}_{{\rm{Na}}})({R}_{{\rm{O}}}+{R}_{{\rm{Na}}})}{2{m}_{{\rm{M}}}^{-1}{R}_{{\rm{M}}}{R}_{{\rm{O}}}+\mathrm{2(}{m}_{{\rm{O}}}^{-1}{R}_{{\rm{O}}}+{m}_{{\rm{M}}}^{-1}{R}_{{\rm{M}}}){R}_{{\rm{M}}}}-\tfrac{2}{3}}{\sqrt{\{{(\tfrac{2{R}_{{\rm{O}}}}{{a}_{0}})}^{2}-\tfrac{1}{3}\}\{{(\tfrac{{R}_{{\rm{O}}}+{R}_{{\rm{Na}}}}{{a}_{0}})}^{2}-\tfrac{1}{3}\}}+2\{{(\tfrac{{R}_{{\rm{O}}}+{R}_{{\rm{Na}}}}{{a}_{0}})}^{2}-\tfrac{1}{3}\}},\end{array}$$

where ${a}_{0}\,=\,2\sqrt{2{R}_{{\rm{O}}}{R}_{{\rm{M}}}+{R}_{{\rm{O}}}^{2}}$. The hard-sphere model examined in this paper gives intuitive and easy comprehension of the thermal expansion behavior of the layered oxides. The density-functional-thery (DFT) calculation successfully reproduces the linear thermal expansion coefficients of several materials such as Al^75^, S^76^, 4d transition metals^77,78^, Os^78^, MgO^79^, CaO^79^, and ZnO^80^, which is beyond the scope of this paper.

## Conclusion

We systematically determined the temperature dependent lattice constant and *z*-coordinates of P2- and O3-type NaMO_2_. We proposed a simple hard-sphere model with constraint that M, upper and lower oxygens are coroneted to each others. The model quantitatively reproduced *a*, *c*, *z*, *α*_*a*_ and *α*_*c*_ for O3-type Na_*x*_MO_2_. On the other hand, *z* coordinate of P2-type Na_*x*_MO_2_ deviates from the hard-sphere model possibly due to Na vacancies. This simple model can be easily applied for the other layered compounds to intuitively understand and design the thermal expansion behaviors.

## Methods

### Sample preparation

Powders of O3- and P2-Na_*x*_MO_2_ (M: transition metal) were synthesized by using conventional solid state reaction. For O3-Na_1.00_CoO_2_, Na_2_O_2_ and Co_3_O_4_ were mixed under the molar ratio of Na:Co = 1.1:1, and calcined at 550°C in O_2_ for 16 h. Then, the product was finely ground, and again calcined in the same condition (this process was repeated once again.). For O3-Na_0.98_Fe_0.5_Co_0.5_O_2_, Na_2_CO_3_, Fe_3_O_4_ and Co_3_O_4_ were mixed under the molar ratio of Na:Fe:Co = 1.05:0.5:0.5, and calcined at 900 °C in air for 15 h. For O3-Na_0.99_Fe_0.5_Ni_0.5_O_2_, Na_2_O_2_, Fe_2_O_3_ and NiO were mixed under the molar ratio of Na:Fe:Ni = 1.2:0.5:0.5, and calcined at 650°C in O_2_ for 15 h. Then the product was finely ground and again calcined in the same condition. For O3-Na_0.94_Ti_0.5_Ni_0.5_O_2_, Na_2_CO_3_, TiO_2_ and NiO were mixed under the molar ratio of Na:Ti:Ni = 1.05:0.5:0.5, and calcined at 900°C in air for 15 h. Then the product was finely ground and again calcined in the same condition.

For P2-Na_0.50_Mn_0.5_Co_0.5_O_2_, Na_2_CO_3_, MnCO_3_ and Co_3_O_4_ were mixed under the molar ratio of Na:Mn:Co = 0.7:0.5:0.5, and calcined at 900°C in air for 12 h, Then, the product was finely ground and again calcined in the same condition. For P2-Na_0.70_Ni_0.33_Mn_0.67_O_2_, Na_2_CO_3_, NiO and Mn_2_O_3_ were mixed in ethanol under the molar ratio of Na:Ni:Mn = 0.7:0.33:0.67, and calcined at 900°C in air for 24 h. Then, the product was finely ground and again calcined in the same condition. For P2-Na_0.69_Ni_0.33_Mn_0.5_Ti_0.17_O_2_, Na_2_CO_3_, NiO, Mn_2_O_3_ and TiO_2_ were mixed under the molar ratio of Na:Ni:Mn:Ti = 0.7:0.33:0.5: 0.17, and calcined at 900°C in air for 18 h. For P2-Na_0.70_Ni_0.33_Mn_0.33_Ti_0.34_O_2_, Na_2_CO_3_, NiO, Mn_2_O_3_ and TiO_2_ were mixed under the molar ratio of Na:Ni:Mn:Ti = 0.7:0.33:0.33:0.34, and calcined at 900°C in air for 12 h. For P2-Na_0.48_Mn_0.5_Fe_0.5_O_2_, Na_2_O_2_, Mn_2_O_3_ and Fe_2_O_3_ were mixed under the molar ratio of Na:Mn:Fe = 0.7:0.5:0.5, and calcined at 900°C in air for 12 h. Then, the product was finely ground and again calcined in the same condition. All the samples were taken out from the hot furnace (>200°C), and then immediately transferred into a vacuum desiccator to avoid moisture in air.

### X-ray diffraction

The synchrotron radiation x-ray diffraction (XRD) patterns were measured at BL02B2 beamline^81^ at SPring-8. The capillary was placed on the Debye-Scherrer camera at the beamline. The sample temperature was controlled by blowing a hot N_2_ in the temperature range of 300 K ≤ *T* ≤ 800 K. The XRD patterns were detected with an imaging plate (IP). The exposure time was 5 min. The wavelength of the x-ray was 0.499420 Å for P2-Na_0.48_Mn_0.5_Fe_0.5_O_2_ and P2-Na_0.50_Mn_0.5_Co_0.5_O_2_, and 0.499892 Å for O3-Na_0.98_Fe_0.5_Co_0.5_O_2_ and Na_0.94_Ti_0.5_Ni_0.5_O_2_, and 0.499838 Å for the others. The wavelengths are calibrated by the cell parameter of standard CeO_2_ powders. Crystal structure was analyzed by RIETAN-FP program^82^. Schematic figure of the crystal structure were drawn by VESTA program^83^. All the reflections can be indexed with the O3-type ($R\bar{3}m$) or P2-type (*P*6_3_/*mmc*) structures except for a tiny amount of impurity of O3-type Fe-rich phase for Na_0.99_Fe_0.5_Ni_0.5_O_2_ and NiO for Na_0.94_Ti_0.5_Ni_0.5_O_2_. All the structural parameters against *T* (300 K ≤ *T* ≤ 800 K) were listed in Tables S1–S9. During heating process, any extra impurity peaks were not appeared. We observed no tendency of Na deintercalation due to heating (Fig. S4).

The actual Na concentrations in the compound were determined by the Rietveld refinement based on the synchrotron XRD patterns at 300 K. We note that ref.^45^ reported a consistency of Na contents determined by ICP-AES (Inductively Coupled Plasma Atomic Emission Spectroscopy) and Rietveld refinement using synchrotron x-ray diffraction for P2-Na_*x*_Mn_1/2_Fe_1/2_O_2_ phase.

### Madelung energy calculation

Madelung energy (*E*_ME_) was computed by the MADEL program in the VESTA software using the Fourier method^83^. The site potential *ϕ*_*i*_ is calculated by the formula ${\varphi }_{i}={\sum }_{j}\frac{{g}_{j}{Z}_{j}}{4\pi {\varepsilon }_{0}{l}_{ij}}$, where *g*_*j*_ is the occupancy of the *j* th ion, *Z*_*i*_ is the valence of the *j* th ion, *ε*_0_ is the vacuum permittivity, and *l*_*ij*_ is the distance between ions *i* and *j*. *E*_ME_ is calculated by using the formula ${E}_{{\rm{ME}}}=\frac{1}{2}{\sum }_{i}{\varphi }_{i}{Z}_{i}{W}_{i}$, where *W*_*i*_ is a factor depending on *g*_*i*_ and the number of equivalent atomic positions at the site *i* in the unit cell. For O3-type structure (space group: $R\bar{3}m$), we put +1, +3, and −2 charges on 3*a* Na (0,0,0), 3*b* M (0,0, $\frac{1}{2}$), and 6*c* O (0,0, *z*) sites in stoichiometric NaMO_2_. In the calculation of the P2-type structure (*P*6_3_/*mmc*), we assume a stoichiometric NaMO_2_ with fully occupied 2*d* Na site. We put +*e*, +3*e*, and −2*e* charges on 2*d* Na $(\frac{1}{3},\frac{2}{3},\frac{3}{4})$, 2*a* M (0,0,0), and 4*f* O $(\frac{1}{3},\frac{2}{3},z)$. A radius (*s*) of the hard sphere was set to 0.3 Å, and Fourier coefficients are summed up to 10 Å^−1^ in the reciprocal space.

## Electronic supplementary material

Supplementary information
