# Supplementary material for: Thermal Expansion in Layered NaxMO2
Source: Sci Rep. 2018 Mar 5;8:3988. doi: 10.1038/s41598-018-22279-9 (PMC5838218; doi:10.1038/s41598-018-22279-9)
Supplement: Supplementary file 1 — Supplementary information [file 41598_2018_22279_MOESM1_ESM.pdf]

Supporting information

Wataru Kobayashi[1,2,3*], Ayumu Yanagita[1], Takahiro Akaba[1], Takahiro Shimono[1], Daiki Tanabe[1], and Yutaka Moritomo[1,2,3]

[1]Graduate School of Pure and Applied Sciences, University of Tsukuba, Ibaraki 305-8571, Japan
[2]Division of Physics, Faculty of Pure and Applied Sciences, University of Tsukuba, Ibaraki 305-8571, Japan
[3]Tsukuba Research Center for Energy Materials Science (TREMS), University of Tsukuba, Ibaraki 305-8571, Japan
[*]kobayashi.wataru.gf@u.tsukuba.ac.jp

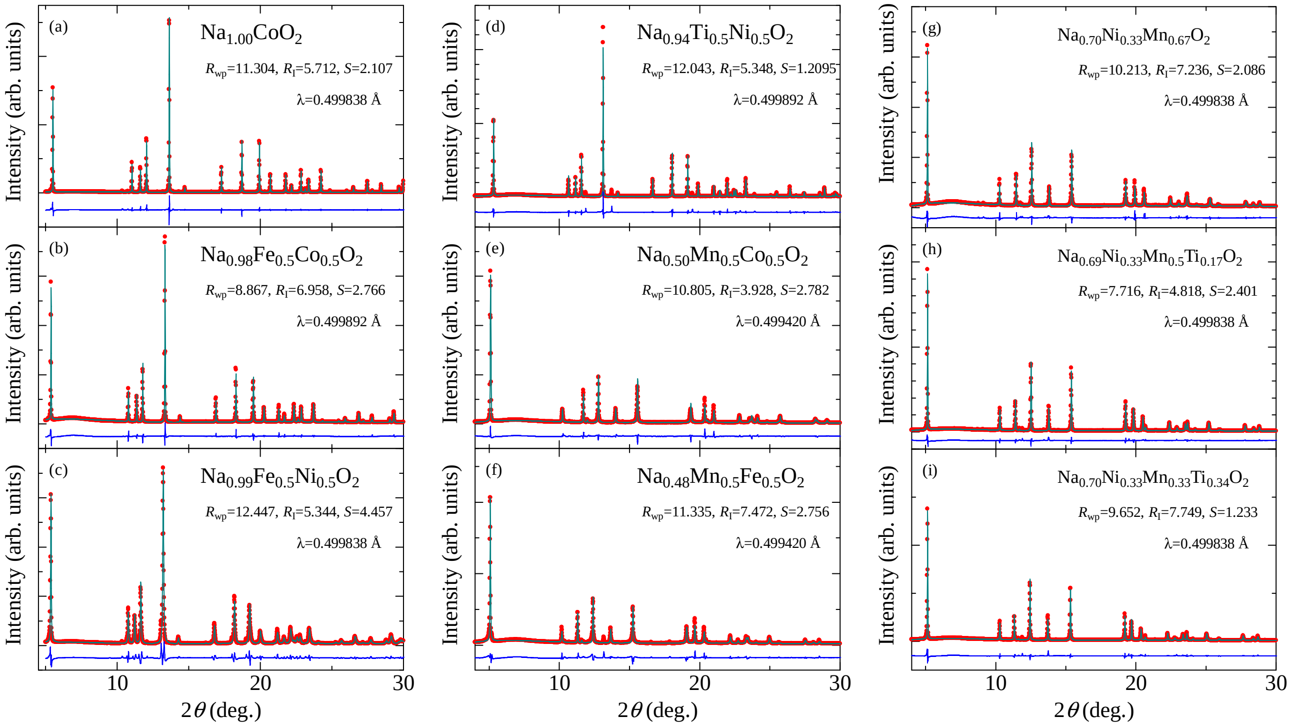

Figure S1. Synchrotron x-ray diffraction patterns of (a-d) O3-type Na_xMO_2 (M=Co, $Fe_{0.5}Co_{0.5}$, $Fe_{0.5}Ni_{0.5}$, and $Ti_{0.5}Ni_{0.5}$) and (e-i) P2-type Na_xMO_2 (M=$Mn_{0.5}Co_{0.5}$, $Mn_{0.5}Fe_{0.5}$, $Ni_{0.33}Mn_{0.67}$, $Ni_{0.33}Mn_{0.5}Ti_{0.17}$, and $Ni_{0.33}Mn_{0.33}Ti_{0.34}$) at 300 K. Red dot, green line, and blue line represent observed intensity (I_{obs}), calculated intensity (I_{cal}), and the difference (I_{obs}-I_{cal}), respectively.

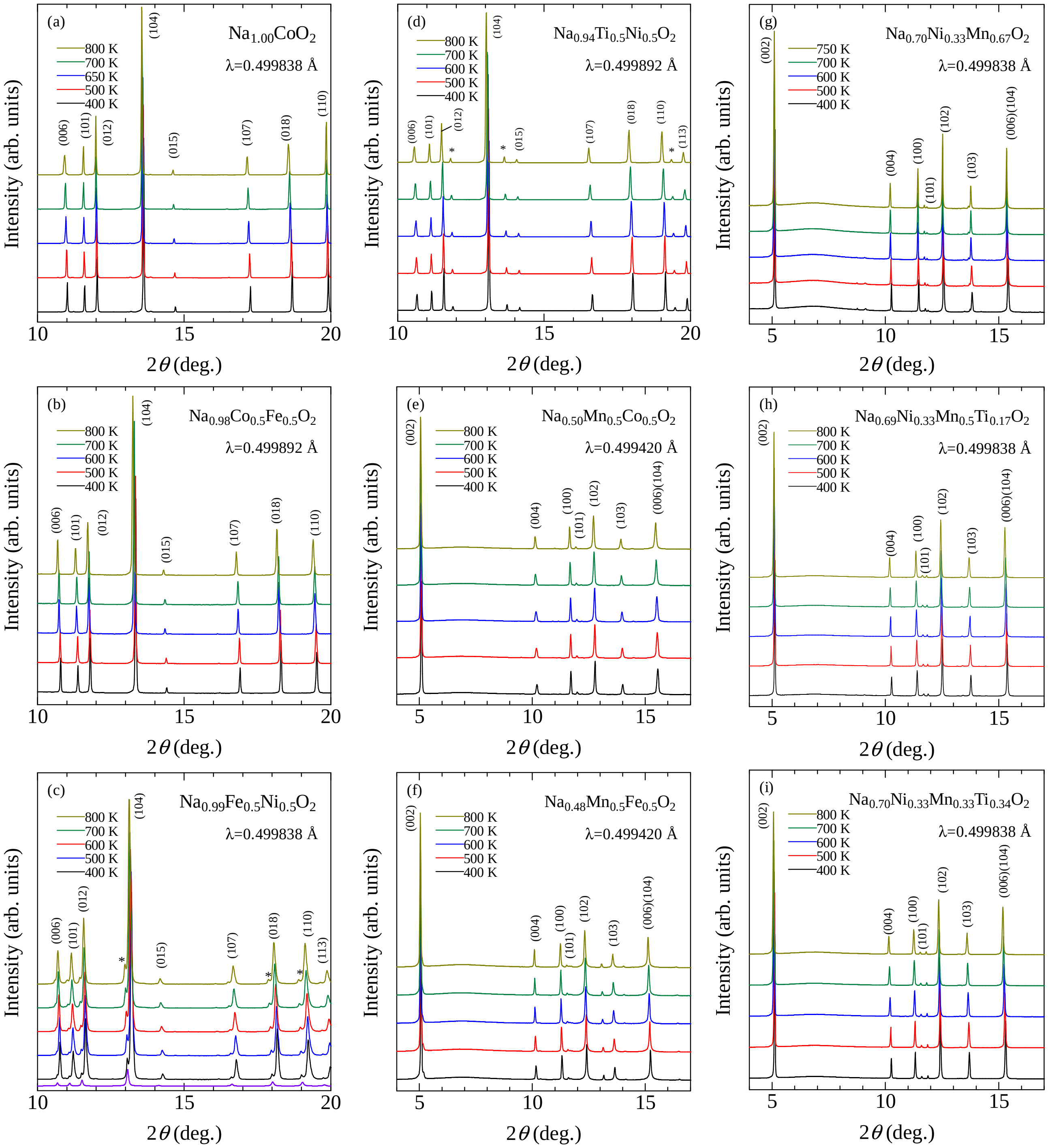

Figure S2. Synchrotron x-ray diffraction patterns of (a-d) O3-type Na_xMO_2 (M=Co, $Fe_{0.5}Co_{0.5}$, $Fe_{0.5}Ni_{0.5}$, and $Ti_{0.5}Ni_{0.5}$) and (e-i) P2-type Na_xMO_2 (M=$Mn_{0.5}Co_{0.5}$, $Mn_{0.5}Fe_{0.5}$, $Ni_{0.33}Mn_{0.67}$, $Ni_{0.33}Mn_{0.5}Ti_{0.17}$, and $Ni_{0.33}Mn_{0.33}Ti_{0.34}$) at 400-800 K. During heating process, extra impurity peaks were not appeared. For $Na_{0.99}Fe_{0.5}Ni_{0.5}O_2$, impurity peaks ($*$) were observed, which is assigned to O3-type Fe-rich phase with $a = 3.02$ Å, and $c = 16.09$ Å. (Purple line represents the simulated pattern.) For $Na_{0.94}Ti_{0.5}Ni_{0.5}O_2$, impurity peaks ($*$) of NiO [space group: $R\bar{3}m$, $a = 2.95$ Å, and $c = 7.23$ Å referred from Rodic, D., Spasojevic, V., Kusigerski, V., Tellgren, R., & Rundlof, H. Magnetic Ordering in Polycrystalline $Ni_xZn_{1-x}O$ Solid Solutions. Phys. Stat. Sol. (b) 218, 527-536 (2000.] were observed.

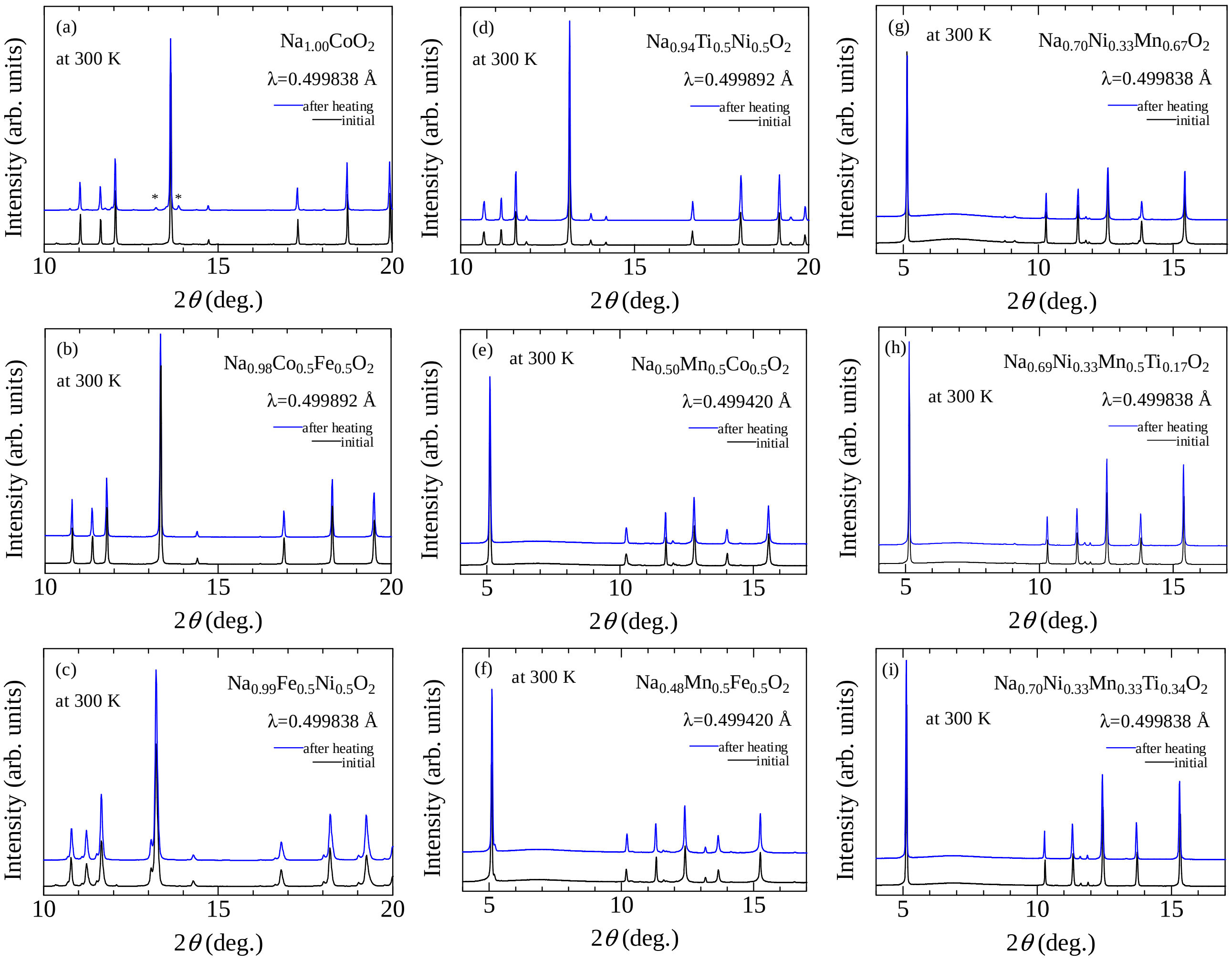

Figure S3. Synchrotron x-ray diffraction patterns of (a-d) O3-type Na_xMO_2 (M=Co, $Fe_{0.5}Co_{0.5}$, $Fe_{0.5}Ni_{0.5}$, and $Ti_{0.5}Ni_{0.5}$) and (e-i) P2-type Na_xMO_2 (M=$Mn_{0.5}Co_{0.5}$, $Mn_{0.5}Fe_{0.5}$, $Ni_{0.33}Mn_{0.67}$, $Ni_{0.33}Mn_{0.5}Ti_{0.17}$, and $Ni_{0.33}Mn_{0.33}Ti_{0.34}$) at 300 K. Black and blue lines represent the initial data and remeasured data after heating process. For $Na_{1.00}CoO_2$, unidentified impurity peaks ($*$) were appeared after heating process.

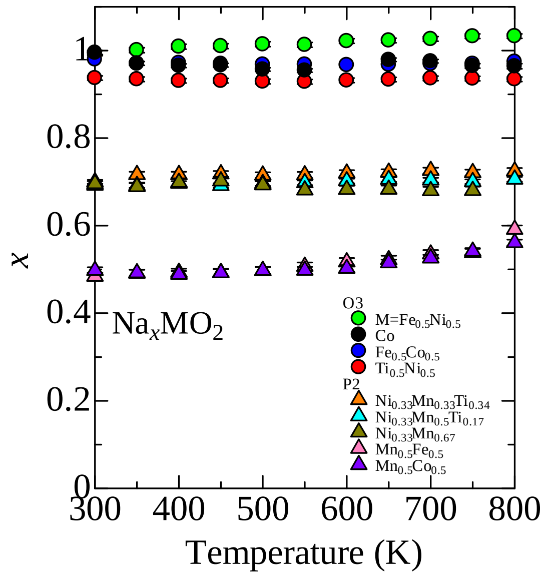

Figure S4. Na concentration (x) against temperature (T) for O3-type Na_xMO_2 (M=Co, $Fe_{0.5}Co_{0.5}$, $Fe_{0.5}Ni_{0.5}$, and $Ti_{0.5}Ni_{0.5}$) and P2-type Na_xMO_2 (M=$Mn_{0.5}Co_{0.5}$, $Mn_{0.5}Fe_{0.5}$, $Ni_{0.33}Mn_{0.67}$, $Ni_{0.33}Mn_{0.5}Ti_{0.17}$, and $Ni_{0.33}Mn_{0.33}Ti_{0.34}$). For O3-type compounds, x is the occupancy (g) of Na site. For P2-type compounds, x is a sum of the occupancies (g_1 and g_2) of Na1 and Na2 sites.

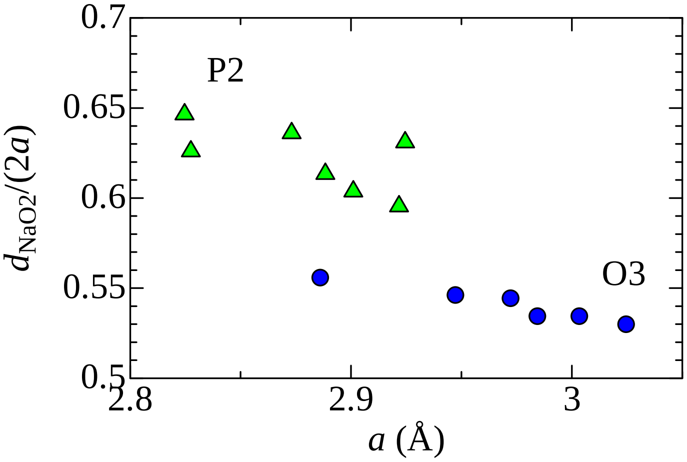

Figure S5. A ratio of a half thickness of NaO_2 layer (d_{NaO_2}) to a-axis lattice constant (a) against a for both P2-type and O3-type compounds at 300 K.

T	a (Å)	c (Å)	z	U_{Na} (Å^2)	U_M (Å^2)	U_O (Å^2)	R_{wp} (%)	R_I (%)	S
300 K	2.88627(2)	15.58680(12)	0.23046(9)	0.00768(40)	0.00249(11)	0.00072(24)	11.3	5.7	2.11
350 K	2.88753(2)	15.59960(12)	0.23064(9)	0.00814(33)	0.00304(11)	0.00169(27)	11.0	5.7	2.07
400 K	2.88892(2)	15.61274(13)	0.23054(10)	0.00871(34)	0.00315(11)	0.00359(32)	11.0	5.2	2.08
450 K	2.89016(2)	15.62555(12)	0.23039(10)	0.00906(35)	0.00267(11)	0.00499(35)	11.1	5.7	2.10
500 K	2.89170(2)	15.64019(12)	0.23008(10)	0.00953(33)	0.00250(11)	0.00616(34)	10.4	5.6	1.98
550 K	2.89318(2)	15.65540(13)	0.22990(10)	0.01013(33)	0.00275(11)	0.00681(35)	10.1	5.3	1.94
600 K	-	-	-	-	-	-	-	-	-
650 K	2.89599(2)	15.68642(14)	0.22970(10)	0.01250(36)	0.00348(11)	0.00737(36)	10.0	5.5	1.92
700 K	2.89776(2)	15.70470(15)	0.22971(10)	0.01446(38)	0.00395(12)	0.00849(39)	10.1	5.7	1.95
750 K	2.89868(3)	15.73302(20)	0.22979(12)	0.01799(51)	0.00500(16)	0.01077(51)	12.3	6.2	2.39
800 K	2.90015(4)	15.75554(25)	0.23005(15)	0.02034(64)	0.00613(21)	0.01179(63)	14.3	7.0	2.80

Table S1. Structural parameters of O3-type $Na_{1.00}CoO_2$ with $R\bar{3}m$ ($Z = 3$) model. Atomic positions are 3a Na (0,0,0), 3b M=Co (0,0,$\frac{1}{2}$), and 6c O (0,0,z). g is occupancy of the Na site. g [=0.995(4)] at 300 K was defined as x. U is isotropic atomic displacement parameter. R_{wp}, R_I, and S are reliable parameters.

T	a (Å)	c (Å)	z	U_{Na} (Å^2)	U_M (Å^2)	U_O (Å^2)	R_{wp} (%)	R_I (%)	S
300 K	2.94748(3)	15.93845(23)	0.23238(12)	0.00923(55)	0.00277(16)	0.00410(44)	8.9	7.0	2.77
350 K	2.94663(3)	15.93754(22)	0.23225(11)	0.01025(45)	0.00289(16)	0.00435(44)	7.5	7.9	2.37
400 K	2.94804(3)	15.95012(23)	0.23215(12)	0.01103(47)	0.00333(17)	0.00500(46)	7.5	8.1	2.38
450 K	2.94944(3)	15.96276(23)	0.23205(12)	0.01214(49)	0.00400(18)	0.00542(48)	7.6	8.2	2.41
500 K	2.95085(4)	15.97478(24)	0.23198(12)	0.01273(50)	0.00453(19)	0.00565(49)	7.6	8.3	2.42
550 K	2.95237(4)	15.98692(24)	0.23196(12)	0.01324(51)	0.00488(19)	0.00579(50)	7.6	8.5	2.43
600 K	2.95395(4)	15.99971(25)	0.23192(12)	0.01432(52)	0.00541(20)	0.00667(52)	7.5	8.4	2.42
650 K	2.95570(4)	16.01448(26)	0.23189(12)	0.01575(54)	0.00600(20)	0.00733(53)	7.4	8.7	2.40
700 K	2.95746(4)	16.02937(26)	0.23194(12)	0.01702(55)	0.00619(21)	0.00808(54)	7.4	8.5	2.39
750 K	2.95964(4)	16.04607(28)	0.23199(13)	0.01833(58)	0.00652(22)	0.00847(56)	7.5	8.7	2.46
800 K	2.96243(4)	16.06386(29)	0.23210(13)	0.01982(60)	0.00714(23)	0.00899(58)	7.5	8.7	2.48

Table S2. Structural parameters of O3-type $Na_{0.98}Fe_{0.5}Co_{0.5}O_2$ with $R\bar{3}m$ ($Z = 3$) model. Atomic positions are 3a Na (0,0,0), 3b M=$Fe_{0.5}Co_{0.5}$ (0,0,$\frac{1}{2}$), and 6c O (0,0,z). g is occupancy of the Na site. g [=0.980(5)] at 300 K was defined as x. U is isotropic atomic displacement parameter. R_{wp}, R_I, and S are reliable parameters.

T	a (Å)	c (Å)	z	U_{Na} (Å^2)	U_M (Å^2)	U_O (Å^2)	R_{wp} (%)	R_I (%)	S
300 K	2.98463(9)	15.94545(63)	0.23334(17)	0.00924(81)	0.00342(25)	0.00934(69)	12.4	5.3	4.46
350 K	2.98673(9)	15.95952(58)	0.23338(16)	0.00920(60)	0.00448(22)	0.00902(65)	12.1	5.5	4.35
400 K	2.98807(9)	15.97262(58)	0.23334(16)	0.00929(62)	0.00508(26)	0.00950(67)	12.0	5.4	4.34
450 K	2.98950(9)	15.98612(59)	0.23329(16)	0.00953(63)	0.00526(27)	0.00957(68)	12.0	5.8	4.34
500 K	2.99106(9)	15.99943(60)	0.23334(16)	0.01043(64)	0.00556(27)	0.01047(69)	11.8	5.8	4.29
550 K	2.99270(9)	16.01358(61)	0.23331(16)	0.01135(64)	0.00582(27)	0.01131(70)	11.6	5.8	4.26
600 K	2.99437(9)	16.02804(61)	0.23346(15)	0.01236(64)	0.00605(27)	0.01162(69)	11.3	5.8	4.16
650 K	2.99627(9)	16.04289(61)	0.23345(15)	0.01363(65)	0.00633(27)	0.01235(69)	11.0	5.9	4.04
700 K	2.99878(9)	16.05649(61)	0.23357(15)	0.01476(65)	0.00682(27)	0.01336(70)	10.7	5.8	3.99
750 K	3.00169(9)	16.06661(59)	0.23366(14)	0.01499(63)	0.00736(27)	0.01456(69)	10.3	5.8	3.87
800 K	3.00469(9)	16.07768(59)	0.23373(14)	0.01541(63)	0.00809(28)	0.01549(69)	10.1	6.1	3.83

Table S3. Structural parameters of O3-type $Na_{0.99}Fe_{0.5}Ni_{0.5}O_2$ with $R\bar{3}m$ ($Z = 3$) model. Atomic positions are $3a$ Na (0,0,0), $3b$ M=$Fe_{0.5}Ni_{0.5}$ $(0,0,\frac{1}{2})$, and $6c$ O $(0,0,z)$. g is occupancy of the Na site. g [=0.994(6)] at 300 K was defined as x. U is isotropic atomic displacement parameter. R_{wp}, R_I, and S are reliable parameters.

T	a (Å)	c (Å)	z	U_{Na} (Å^2)	U_M (Å^2)	U_O (Å^2)	R_{wp} (%)	R_I (%)	S
300 K	3.00358(4)	16.12273(24)	0.23382(13)	0.00805(62)	0.00525(21)	0.00632(51)	12.0	5.3	1.21
350 K	3.00413(4)	16.13057(26)	0.23379(14)	0.00846(55)	0.00629(24)	0.00612(55)	11.1	4.8	1.31
400 K	3.00582(4)	16.14406(26)	0.23366(14)	0.00977(57)	0.00675(25)	0.00676(56)	11.0	4.7	1.30
450 K	3.00749(4)	16.15738(26)	0.23370(14)	0.01109(59)	0.00752(26)	0.00754(58)	10.9	4.7	1.30
500 K	3.00910(5)	16.17074(28)	0.23364(14)	0.01261(62)	0.00796(27)	0.00800(60)	11.0	4.7	1.31
550 K	3.01091(5)	16.18490(29)	0.23360(14)	0.01388(65)	0.00876(29)	0.00868(62)	10.9	5.3	1.31
600 K	3.01291(5)	16.19905(29)	0.23368(14)	0.01513(64)	0.00965(28)	0.00988(62)	10.3	4.8	1.25
650 K	3.01519(5)	16.21410(30)	0.23364(14)	0.01642(64)	0.01039(29)	0.01101(63)	10.0	4.8	1.22
700 K	3.01753(5)	16.22830(32)	0.23358(14)	0.01793(66)	0.01108(30)	0.01181(64)	9.8	4.9	1.20
750 K	3.02019(5)	16.24450(33)	0.23360(14)	0.01919(68)	0.01184(31)	0.01285(66)	9.7	4.6	1.20
800 K	3.02287(5)	16.26133(33)	0.23365(14)	0.02046(69)	0.01261(32)	0.01382(68)	9.6	4.5	1.21

Table S4. Structural parameters of O3-type $Na_{0.94}Ti_{0.5}Ni_{0.5}O_2$ with $R\bar{3}m$ ($Z = 3$) model. Atomic positions are $3a$ Na (0,0,0), $3b$ M=$Ti_{0.5}Ni_{0.5}$ $(0,0,\frac{1}{2})$, and $6c$ O $(0,0,z)$. g is occupancy of the Na site. g [=0.937(5)] at 300 K was defined as x. U is isotropic atomic displacement parameter. R_{wp}, R_I, and S are reliable parameters.

T	a (Å)	c (Å)	z	g_2	U_{Na} (Å^2)	U_M (Å^2)	U_O (Å^2)	R_{wp} (%)	R_I (%)	S
300 K	2.82459(5)	11.20617(33)	0.08704(24)	0.2204(49)	0.0256(28)	0.00293(16)	0.00696(70)	10.8	3.9	2.78
350 K	2.82558(6)	11.21850(37)	0.08630(23)	0.2167(33)	0.0284(23)	0.00320(16)	0.00590(69)	10.8	4.1	2.79
400 K	2.82675(6)	11.23167(38)	0.08633(23)	0.2140(33)	0.0328(24)	0.00370(17)	0.00570(70)	10.6	3.4	2.75
450 K	2.82795(6)	11.24451(39)	0.08628(23)	0.2164(34)	0.0380(26)	0.00434(17)	0.00583(71)	10.6	3.8	2.73
500 K	2.82921(6)	11.25826(40)	0.08597(23)	0.2140(35)	0.0413(27)	0.00461(18)	0.00605(74)	10.6	3.9	2.77
550 K	2.83047(6)	11.27160(40)	0.08585(23)	0.2135(35)	0.0424(28)	0.00488(18)	0.00678(75)	10.5	4.1	2.74
600 K	2.83200(6)	11.28519(42)	0.08557(23)	0.2136(34)	0.0448(28)	0.00524(18)	0.00851(78)	10.2	4.1	2.70
650 K	2.83372(6)	11.29714(39)	0.08556(22)	0.2127(33)	0.0474(28)	0.00568(18)	0.00931(77)	9.8	4.3	2.59
700 K	2.83575(6)	11.30686(38)	0.08560(21)	0.2125(31)	0.0478(26)	0.00591(17)	0.00949(74)	9.3	4.3	2.47
750 K	2.83805(5)	11.31410(37)	0.08605(20)	0.2125(30)	0.0471(25)	0.00622(17)	0.00979(72)	8.8	4.6	2.38
800 K	2.84061(5)	11.32068(37)	0.08629(20)	0.2137(29)	0.0478(25)	0.00666(17)	0.01003(71)	8.6	4.6	2.32

Table S5. Structural parameters of P2-type $Na_{0.50}Mn_{0.5}Co_{0.5}O_2$ with $P6_3/mmc$ ($Z = 2$) model. Atomic positions are $2a$ M=$Mn_{0.5}Co_{0.5}$ (0,0,0), $4f$ O ($\frac{1}{3}$,$\frac{2}{3}$,z), $2b$ Na2 (0,0,$\frac{1}{4}$), and $2d$ Na1 ($\frac{1}{3}$,$\frac{2}{3}$,$\frac{3}{4}$). g_2 (g_1) is occupancy of the Na2 (Na1) site. $g_1 + g_2$ [=0.498(3)] at 300 K was defined as x. g_1 was fixed at $x - g_2$. U is isotropic atomic displacement parameter. A constrained condition of $U_{Na} = U_{Na1} = U_{Na2}$ was set. R_{wp}, R_I, and S are reliable parameters.

T	a (Å)	c (Å)	z	g_2	U_{Na} (Å^2)	U_M (Å^2)	U_O (Å^2)	R_{wp} (%)	R_I (%)	S
300 K	2.88835(5)	11.15881(29)	0.09120(28)	0.2626(41)	0.0436(24)	0.00361(19)	0.00750(78)	10.2	7.2	2.09
350 K	2.88755(5)	11.16493(32)	0.09100(31)	0.2681(44)	0.0480(28)	0.04803(23)	0.00895(91)	9.4	8.5	3.01
400 K	2.88873(5)	11.17524(31)	0.09082(31)	0.2677(44)	0.0510(29)	0.00509(23)	0.00903(91)	9.3	8.8	2.99
450 K	2.88995(5)	11.18465(30)	0.09088(31)	0.2680(44)	0.0544(30)	0.00518(22)	0.00878(89)	9.1	9.3	2.95
500 K	2.89113(5)	11.19317(28)	0.09076(30)	0.2705(44)	0.0569(30)	0.00522(21)	0.00905(87)	8.9	8.8	2.88
550 K	2.89252(4)	11.20122(25)	0.09035(28)	0.2716(42)	0.0575(29)	0.00511(20)	0.00828(81)	8.6	8.7	2.79
600 K	2.89402(4)	11.20906(22)	0.09037(28)	0.2686(41)	0.0573(28)	0.00523(19)	0.00868(78)	8.4	9.1	2.72
650 K	2.89555(4)	11.21804(21)	0.09018(27)	0.2659(41)	0.0588(28)	0.00579(19)	0.00941(79)	8.4	9.0	2.72
700 K	2.89708(4)	11.22816(22)	0.09009(28)	0.2655(42)	0.0614(29)	0.00638(20)	0.01030(82)	8.4	9.2	2.71
750 K	2.89899(4)	11.23983(22)	0.08990(28)	0.2588(43)	0.0662(30)	0.00704(21)	0.01053(83)	8.3	9.3	2.68
800 K	-	-	-	-	-	-	-	-	-	-

Table S6. Structural parameters of P2-type $Na_{0.70}Ni_{0.33}Mn_{0.67}O_2$ with $P6_3/mmc$ ($Z = 2$) model. Atomic positions are $2a$ M=$Ni_{0.33}Mn_{0.67}$ (0,0,0), $4f$ O ($\frac{1}{3}$,$\frac{2}{3}$,z), $2b$ Na2 (0,0,$\frac{1}{4}$), and $2d$ Na1 ($\frac{1}{3}$,$\frac{2}{3}$,$\frac{3}{4}$). g_2 (g_1) is occupancy of the Na2 (Na1) site. $g_1 + g_2$ [=0.696(4)] at 300 K was defined as x. g_1 was fixed at $x - g_2$. U is isotropic atomic displacement parameter. A constrained condition of $U_{Na} = U_{Na1} = U_{Na2}$ was set. R_{wp}, R_I, and S are reliable parameters.

T	a (Å)	c (Å)	z	g_2	U_{Na} (Å^2)	U_M (Å^2)	U_O (Å^2)	R_{wp} (%)	R_I (%)	S
300 K	2.90101(3)	11.12868(16)	0.09263(17)	0.2292(25)	0.0370(12)	0.00430(12)	0.00879(43)	7.7	4.8	2.40
350 K	2.90222(3)	11.14286(17)	0.09274(17)	0.2341(25)	0.0393(13)	0.00483(13)	0.00875(45)	7.8	4.7	2.44
400 K	2.90380(3)	11.15635(17)	0.09257(17)	0.2358(25)	0.0417(13)	0.00546(13)	0.00898(45)	7.6	4.8	2.37
450 K	2.90525(3)	11.16788(16)	0.09233(17)	0.2367(25)	0.0447(14)	0.00607(14)	0.00954(46)	7.6	5.4	2.40
500 K	2.90684(3)	11.17941(17)	0.09239(17)	0.2356(25)	0.0459(14)	0.00655(14)	0.00983(47)	7.6	5.7	2.41
550 K	2.90858(3)	11.19157(17)	0.09229(17)	0.2357(25)	0.0480(14)	0.00702(14)	0.01033(48)	7.5	5.8	2.38
600 K	2.91030(3)	11.20366(18)	0.09232(17)	0.2365(25)	0.0503(15)	0.00760(14)	0.01077(49)	7.4	5.9	2.37
650 K	2.91216(3)	11.21674(18)	0.09241(17)	0.2372(25)	0.0523(15)	0.00825(15)	0.01124(50)	7.3	5.8	2.35
700 K	2.91420(4)	11.23078(19)	0.09225(17)	0.2366(26)	0.0553(16)	0.00904(16)	0.01192(52)	7.3	5.9	2.37
750 K	2.91629(4)	11.24461(20)	0.09206(17)	0.2376(26)	0.0576(16)	0.00977(16)	0.01232(53)	7.2	6.1	2.37
800 K	2.91830(4)	11.25943(20)	0.09208(18)	0.2355(26)	0.0606(17)	0.01057(17)	0.01292(55)	7.2	6.2	2.39

Table S7. Structural parameters of P2-type $Na_{0.69}Ni_{0.33}Mn_{0.5}Ti_{0.17}O_2$ with $P6_3/mmc$ ($Z = 2$) model. Atomic positions are $2a$ M=$Ni_{0.33}Mn_{0.5}Ti_{0.17}$ (0,0,0), $4f$ O ($\frac{1}{3}$,$\frac{2}{3}$,z), $2b$ Na2 (0,0,$\frac{1}{4}$), and $2d$ Na1 ($\frac{1}{3}$,$\frac{2}{3}$,$\frac{3}{4}$). g_2 (g_1) is occupancy of the Na2 (Na1) site. $g_1 + g_2$ [=0.692(3)] at 300 K was defined as x. g_1 was fixed at $x - g_2$. U is isotropic atomic displacement parameter. A constrained condition of $U_{Na} = U_{Na1} = U_{Na2}$ was set. R_{wp}, R_I, and S are reliable parameters.

T	a (Å)	c (Å)	z	g_2	U_{Na} (Å^2)	U_M (Å^2)	U_O (Å^2)	R_{wp} (%)	R_I (%)	S
300 K	2.92173(4)	11.13896(22)	0.09381(21)	0.2189(30)	0.0308(15)	0.00520(18)	0.00718(54)	9.7	7.7	1.23
350 K	2.92330(4)	11.15429(22)	0.09379(22)	0.2197(31)	0.0340(15)	0.00590(19)	0.00799(56)	9.6	7.9	1.23
400 K	2.92482(4)	11.16938(23)	0.09363(22)	0.2236(31)	0.0371(16)	0.00664(20)	0.00856(58)	9.6	8.1	1.24
450 K	2.92633(5)	11.18483(24)	0.09348(22)	0.2265(32)	0.0402(17)	0.00714(21)	0.00939(62)	9.7	8.5	1.26
500 K	2.92835(5)	11.20161(24)	0.09309(22)	0.2280(32)	0.0425(17)	0.00779(21)	0.01016(63)	9.5	8.4	1.23
550 K	2.92985(5)	11.21612(25)	0.09311(23)	0.2275(33)	0.0447(18)	0.00813(22)	0.01048(65)	9.6	8.6	1.25
600 K	2.93169(5)	11.23004(25)	0.09305(22)	0.2281(32)	0.0475(19)	0.00846(22)	0.01034(65)	9.4	8.5	1.22
650 K	2.93383(5)	11.24369(25)	0.09293(22)	0.2310(32)	0.0494(19)	0.00899(22)	0.01048(64)	9.1	8.4	1.18
700 K	2.93595(5)	11.25683(27)	0.09290(22)	0.2307(33)	0.0519(20)	0.00969(24)	0.01066(67)	9.2	8.7	1.21
750 K	2.93851(5)	11.27133(28)	0.09259(22)	0.2281(33)	0.0552(21)	0.01064(25)	0.01140(70)	9.2	8.9	1.20
800 K	2.94095(5)	11.28670(29)	0.09253(23)	0.2303(34)	0.0572(21)	0.01136(25)	0.01156(71)	9.1	9.0	1.20

Table S8. Structural parameters of P2-type $Na_{0.70}Ni_{0.33}Mn_{0.33}Ti_{0.34}O_2$ with $P6_3/mmc$ ($Z = 2$) model. Atomic positions are $2a$ M=$Ni_{0.33}Mn_{0.33}Ti_{0.34}$ (0,0,0), $4f$ O ($\frac{1}{3}$,$\frac{2}{3}$,z), $2b$ Na2 (0,0,$\frac{1}{4}$), and $2d$ Na1 ($\frac{1}{3}$,$\frac{2}{3}$,$\frac{3}{4}$). g_2 (g_1) is occupancy of the Na2 (Na1) site. $g_1 + g_2$ [=0.700(3)] at 300 K was defined as x. g_1 was fixed at $x - g_2$. U is isotropic atomic displacement parameter. A constrained condition of $U_{Na} = U_{Na1} = U_{Na2}$ was set. R_{wp}, R_I, and S are reliable parameters.

T	a (Å)	c (Å)	z	g_2	U_{Na} (Å^2)	U_M (Å^2)	U_O (Å^2)	R_{wp} (%)	R_I (%)	S
300 K	2.92452(8)	11.24690(48)	0.08592(32)	0.1927(57)	0.0137(32)	0.00354(25)	0.0092(9)	11.3	7.5	2.76
350 K	2.92554(8)	11.26275(48)	0.08571(29)	0.1961(37)	0.0172(26)	0.00459(25)	0.0095(9)	11.1	7.0	2.69
400 K	2.92670(7)	11.27969(47)	0.08513(28)	0.1959(36)	0.0219(27)	0.00565(25)	0.0108(9)	10.8	6.2	2.63
450 K	2.92860(7)	11.29240(47)	0.08471(28)	0.1969(36)	0.0254(29)	0.00645(26)	0.0117(10)	10.8	6.4	2.63
500 K	2.93092(7)	11.30330(47)	0.08429(28)	0.1959(37)	0.0267(29)	0.00711(27)	0.0121(10)	10.8	6.3	2.62
550 K	2.93328(7)	11.31396(47)	0.08432(29)	0.1976(37)	0.0269(30)	0.00766(28)	0.0127(10)	10.8	6.4	2.64
600 K	2.93564(7)	11.32414(48)	0.08424(29)	0.1963(37)	0.0308(32)	0.00825(29)	0.0136(10)	10.8	6.7	2.64
650 K	2.93812(8)	11.33443(49)	0.08441(29)	0.1929(37)	0.0315(32)	0.00908(30)	0.0146(11)	10.7	7.1	2.65
700 K	2.94066(7)	11.34432(47)	0.08422(28)	0.1927(36)	0.0331(32)	0.00992(30)	0.0157(11)	10.4	7.1	2.59
750 K	2.94316(7)	11.35386(48)	0.08419(28)	0.1933(36)	0.0324(32)	0.01064(31)	0.0166(11)	10.3	7.3	2.59
800 K	2.94609(7)	11.36259(46)	0.08586(28)	0.1897(34)	0.0291(30)	0.11220(31)	0.0189(11)	10.0	7.8	2.51

Table S9. Structural parameters of P2-type $Na_{0.48}Mn_{0.5}Fe_{0.5}O_2$ with $P6_3/mmc$ ($Z = 2$) model. Atomic positions are $2a$ M=$Mn_{0.5}Fe_{0.5}$ (0,0,0), $4f$ O ($\frac{1}{3}$,$\frac{2}{3}$,z), $2b$ Na2 (0,0,$\frac{1}{4}$), and $2d$ Na1 ($\frac{1}{3}$,$\frac{2}{3}$,$\frac{3}{4}$). g_2 (g_1) is occupancy of the Na2 (Na1) site. $g_1 + g_2$ [=0.484(4)] at 300 K was defined as x. g_1 was fixed at $x - g_2$. U is isotropic atomic displacement parameter. A constrained condition of $U_{Na} = U_{Na1} = U_{Na2}$ was set. R_{wp}, R_I, and S are reliable parameters.
